# The effects of blood group types on the risk of COVID-19 infection and its clinical outcome

**DOI:** 10.3906/sag-2005-395

**Published:** 2020-06-23

**Authors:** Hakan GÖKER, Elifcan ALADAĞ-KARAKULAK, Haluk DEMİROĞLU, Cağlayan Merve AYAZ, Yahya BÜYÜKAŞIK, Ahmet Cağkan İNKAYA, Salih AKSU, Nilgün SAYINALP, İbrahim C. HAZNEDAROĞLU, Ömrüm UZUN, Murat AKOVA, Osman ÖZCEBE, Serhat ÜNAL

**Affiliations:** 1 Department of Internal Medicine, Section of Hematology,Faculty of Medicine, Hacettepe University, Ankara Turkey; 2 Department of Infectious Diseases, Faculty of Medicine, Hacettepe University, Ankara Turkey

**Keywords:** Blood groups, COVID-19, SARS-COV-2, susceptibility, clinical outcome

## Abstract

**Background/aim:**

COVID-19 (Coronavirus disease of 2019) is an infectious disease outbreak later on declared as a pandemic, caused by the SARS-CoV-2 (severe acute respiratory syndrome coronavirus-2). It spreads very rapidly and can result in severe acute respiratory failure. The clinical studies have shown that advanced age and chronic diseases increase the risk of infection. However, influence of the blood groups on COVID-19 infection and its outcome remains to be confirmed. The aim of this study is to investigate whether there exists a relationship between the blood groups of the patients and risk of SARS-CoV-2 infection and the clinical outcomes in COVID-19 patients.

**Material and method:**

186 patients with PCR confirmed diagnosis of COVID-19 were included in this study. Age, sex, blood groups, comorbidities, need for intubation and intensive care unit follow up and mortalities of the patients were analyzed retrospectively. 1881 healthy individuals, who presented to the Hacettepe University Blood Bank served as the controls.

**Results:**

The most frequently detected blood group was blood group A (57%) amongst the COVID-19 patients. This was followed by blood group O (24.8%). The blood group types did not affect the clinical outcomes. The blood group A was statistically significantly more frequent among those infected with COVID-19 compared to controls (57% vs. 38%, P < 0.001; OR: 2.1). On the other hand, the frequency of blood group O was significantly lower in the COVID-19 patients, compared to the control group (24.8% vs. 37.2%, P: 0.001; OR: 1.8).

**Conclusions:**

The results of the present study suggest that while the blood group A might have a role in increased susceptibility to the COVID-19 infection, the blood group O might be somewhat protective. However, once infected, blood group type does not seem to influence clinical outcome.

## 1. Introduction

COVID-19 was first described as a serious infection leading to significant morbidity and mortality in Wuhan, China in January 2020 [1]. World Health Organization (WHO) declared COVID-19 infection caused by SARS-CoV-2 virus as a pandemic on March 2020. SARS-COV-2 is beta coronavirus that is closely related to SARS-CoV, both viruses use the angiotensin-converting enzyme-related carboxypeptidase (ACE2) in order to enter the cell [1,2]. COVID-19 disease has been observed over 5,5 million people worldwide as of May 25, 2020; and it has caused the death over 350,000 of these patients. Many studies have shown that advanced age, male sex, and comorbidities of the person increase the risk and severity of the infection [2]. To date, there is no specific biological marker that has been demonstrated to predict the disease. Many previous studies indicated that the connection between hepatitis B and the Norwalk virus infection with the blood groups [3,4]. Few studies on SARS-CoV-1 demonstrated that there exists a relationship between infection risk and the blood types and that the blood group O was somewhat protective against the SARS-CoV-1 [5,6].

There are only few clinical studies examining the relationship between SARS-CoV-2 and the blood groups. In these studies, it was demonstrated that the blood group O had a negative predictive effect and the blood group A was more frequent in patients who presented with severe pulmonary damage [7–9]. The aim of this study is to investigate the distribution and relationship between the blood groups amongst the COVID-19 patients and their clinical outcomes at a referral university hospital.

## 2. Materials and methods

### 2.1. Patient population

The present study included 207 patients who were followed at Hacettepe University School of Medicine Hospitals between 10/03/2020 and 05/05/2020 with the COVID-19 infection who were positive for the SARS-CoV-2 RNA test through PCR from the nasopharyngeal swab, and who were approached in accordance with the treatment guidelines of the Turkish Ministry of Health. The medical records of patients were retrospectively analysed. The patients, whose blood group analysis that were not done at our hospital, were later on contacted, and their blood groups were also obtained. A total of 186 patients were evaluable for the final statistical analysis. The remaining 21 patients were excluded from the study because their blood group information was not reliable or the patients could not be reached. Clinical data including age, sex, comorbidies, intubation needs, intensive care admissions and outcome of the patients were obtained from medical records.

Due to the existing protocols of the hospitals of Hacettepe University Faculty of Medicine, all of the ethical considerations were strictly followed. As a standard care/action of the hospitals of the Hacettepe University Faculty of Medicine, it has been recognized from the patient records that all of the studied patients gave informed consent at the time of admission to the hospital for diagnostic/therapeutic procedures as standards of care. Local Ethical Committee approval was obtained from Hacettepe University numbered GO 20/434 and additionally, Turkish Health Ministry approval was also obtained on May 4th, 2020 for this study as required.

As for the comorbidities, histories of cardiovascular disease, respiratory disease, obesity, diabetes mellitus, and malignancy were screened. Hypertension, coronary artery disease, congestive heart failure, and arrhythmias were classified as cardiovascular diseases; COPD and asthma were classified as respiratory diseases. Clinical outcomes were determined as the need for intubation, the need for ICU, and death.

In order to determine the normal distribution of the blood groups, 1881 healthy individuals, who applied to the Hacettepe University Blood Bank affiliated with our center between 01/03/2020 and 01/05/2020, were included as the control group. The blood group distributions, age and sex of these patients were recorded. 

### 2.2. Statistical analysis

Analyses were made using SPSS version 25.0 (IBM Corp., Armonk, NY, USA). The distribution between the blood groups of the patient and control groups was evaluated using chi-square and Fisher Exact tests. The impacts of blood groups and comorbidities on the clinical outcomes were evaluated using logistic regression analysis. Hosmer-Lemeshow test was used for goodness-of-fit. The cases with the P-value below 0.05 were accepted as statistically significant. 

## 3. Results

The clinical characteristics and outcomes of 186 patients included in the study according to their blood groups are presented in Tables 1 and 2. The median age of patients was 42 (19–92) and the percentage of female patients was 46.2%. The most frequently detected blood group was blood group A with 57% amongst the COVID-19 patients. This was followed by blood group O with 24.8%. There was a history of cardiovascular disease in 35 patients, obesity in 9 patients, diabetes mellitus in 16 patients, respiratory disease in 14 patients, and malignancy in 10 patients. Of the 10 patients with a history of malignancy, 3 had colon adenocarcinoma, 2 had non-Hodgkin lymphoma, 2 had MDS-RAEB2, 1 had HCC, 1 had bladder adenocarcinoma and 1 had pancreatic adenocarcinoma. 

**Table 1:** The distribution of the blood groups according to the clinical characteristics (CVD: Cardiovascular disease, DM: Diabetes mellitus, RD: Respiratory disease; m: male; f: female).

	n	(m/f) (n/%)	P	Age (median)	P	CVD (n) (n/%)	P	Obesity (n/%)	P	DM (n/%)	P	RD (n/%)	P	Malignancy (n/%)	P
A Non-A	106 80	58/48 (54.7/45.3) 42/38 (52.5/47.5)	0.76	43 (19–84) 41 (20–92)	0.83	20 (18.9) 15 (18.8)	0.98	6 (5.7) 3 (3.8)	0.54	12 (11.3) 4 (5)	0.12	8 (7.5) 6 (7.5)	0.99	7 (6.6) 7 (3.8)	0.39
B Non-B	20 166	9/11 (45/55) 91/75 (54.8/45.2)	0.4	48 (26–92) 42 (19–84)	0.46	4 (20) 31 (18.7)	0.88	2 (10) 7 (4.2)	0.25	0 16 (9.6)	0.14	2 (10) 12 (7.2)	0.65	1 (5) 9 (5.4)	0.93
AB Non-AB	14 172	8/6 (57.1/42.9) 92/80 (53.5/46.5)	0.79	33.5 (20–64) 42.5 (19–92)	0.17	0 35 (20.3)	0.06	0 9 (5.2)	0.38	1 (7.1) 15 (8.7)	0.84	0 14 (8.1)	0.26	1 (7.1) 9 (5.2)	0.76
O Non-O	46 140	25/21 (54.3/45.7) 75/65 (53.6/46.2)	0.92	41 (23–84) 43 (19–92)	0.58	11 (23.9) 24 (17.1)	0.30	1 (2.2) 8 (5.7)	0.33	3 (6.5) 13 (9.3)	0.56	4 (8.7) 10 (7.1)	0.72	1 (2.2) 9 (6.4)	0.26
Rh– Rh+	26 160	15/11 (57.7/42.3) 85/75 (53.1/46.9)	0.66	47 (20–73) 41.5 (19–92)	0.44	3 (11.5) 32 (20)	0.30	2 (7.7) 7 (4.4)	0.46	0 16 (10)	0.09	1 (3.8) 13 (8.1)	0.44	4 (15.4) 6 (3.8)	0.015
Total	186	100/86 (53.8/46.2)		42(19–92)		35		9		16		14		10	

**Table 2:** The distribution of the blood groups according to the outcomes of COVID-19 (ICU: Intensive care unit)

	n	ICU ( n/%)	P	Intubation ( n/%)	P	ex ( n/%)	P
A Non-A	106 80	17 (16) 16 (17.7)	0.76	7 (6.6) 4 (5.1)	0.66	6 (5.7) 3 (3.8)	0.54
B Non-B	20 166	3 (15.8) 28 (16.9)	0.4	0 11 (6.6)	0.24	2(10) 7 (4.2)	0.25
AB Non-AB	14 172	4 (28.6) 27 (15.8)	0.21	1 (7.1) 10 (5.8)	0.84	0 9 (5.2)	0.38
O Non-O	46 140	7 (15.2) 24 (17.3)	0.74	3 (6.5) 8 (5.8)	0.84	1 (2.2) 8 (5.7)	0.33
Rh– Rh+	26 160	6 (23.1) 25 (15.7)	0.35	2 (7.7) 9 (5.7)	0.68	1 (3.8) 8 (5)	0.79
Total	186	31		11		9	

The distribution of the patient and control groups according to their blood groups is presented in Table 3. When the healthy control group was compared to the COVID-19 patient group, it was observed that the COVID-19 infection rate was statistically significantly higher in those with blood group A (57% vs. 38%, P <0.001; OR: 2.1). Though, COVID-19 was significantly more seen with the blood group A, on the other hand, Rh factor did not make any significant difference (P > 0.05)

**Table 3:** The comparison of ABO blood groups in patient and control groups (The reference for each group is all other 3 groups for ABO blood groups) (OR: Odds ratio).

	Patient (n/%)	Control (n/%)	OR	P	95% CI
A	106 (57)	716 (38)	2.1 (1.5–2.9)	<0.001	1.84–1.89
B	20 (10.8)	277(14.7)	1.4 (0.8–2.3)	0.155	1.90–1.96
AB	14 (7.5)	188 (10)	1.3 (0.7–2.3)	0.364	1.89–1.96
O	46 (24.8)	701 (37.2)	1.8 (1.2–2.5)	0.001	1.92–1.95
Rh–	26 (14)	174 (9.2)	ref		ref
Rh+	160 (86)	1708 (90.8)	1.2 (0.9–1.4)	0.321	1.86–1.91

It was observed that the blood group O was significantly lower in the COVID-19 patient group in comparison to the controls (24.8% vs. 37.2%, P: 0.001; OR: 1.8). There was no difference between blood groups of B and AB.

The effect of blood groups on clinical outcomes of COVID-19 patients are presented in Table 4. No significant effect of ABO and Rh systems were demonstrated on the clinical outcomes.

**Table 4:** The effects of blood groups on clinical outcomes of COVID-19.

	intubation		ICU		mortality	
	P	OR	P	OR	P	OR
A vs. non-A	0.66	1.32 (0.37–4)	0.94	1.03 (0.39–2.7)	0.35	2.73 (0.31–23.4)
B vs. non-B	0.99	-	0.96	1.03 (0.23–4.5)	0.19	5.01 (0.42–58.8)
AB vs. non-AB	0.84	1.23 (0.1–10.4)	0.32	2 (0.4–8.5)	0.99	-
O vs. non-O	0.66	1.14 (0.2–4.5)	0.74	1.16 (0.4–2.9)	0.35	2.72 (0.33–22.4)
Rh+ vs. Rh–	0.68	1.38 (0.28–6.8)	0.44	1.49 (0.53–4.1)	0.84	1.23 (0.14–10.5)

## 4. Discussion

A total of 186 COVID-19 patients, who were followed at the Hacettepe University, were included in the present study. It was observed that blood group A was more frequent and the blood group O was less frequent in COVID-19 patients compared to the control group. The blood group A was significantly more frequent and the blood group O was less frequent amongst the COVID-19 patients when compared to the controls (P < 0.001 and P: 0.001, respectively)

When we looked at the effect of blood groups and Rh type on the clinical outcomes, it was demonstrated that the blood groups did not have significant predictive effects on the need for intubation, need for ICU hospitalization and mortality; and that the most significant factors affecting the clinical outcomes were age and the history of comorbidity. Few studies evaluated were evaluated in blood groups in terms of the needs for intubation, ICU hospitalization and clinical outcomes [7,8].

There have been studies indicating the predictive effect of ABO blood groups on the Helicobacter pylori, Norwalk virus, and SARS-CoV [6]. In a previous study, conducted with 42 healthcare professionals in Hong Kong during the SARS epidemic period, it was presented that those with blood group O had a lower incidence of SARS-CoV infection than the non-O (OR, 0.18; 95% confidence interval, 0.04–0.81). The patients with blood group B also demonstrated a tendency to the disease; however, there was no statistical significance (OR: 1.46) [5]. In an animal model, which was carried out to present the mechanism of this result, it was found that the anti-A antibodies found in individuals with blood group O inhibited the interaction of SARS-CoV-1 virus S spike protein and the ACE-2 receptor [10]. 

A number of studies have been carried out on this subject since the beginning of the SARS-CoV-2 pandemic. First of all, in a study on 265 COVID-19 patients, it was presented that the blood group O was less frequent in severe COVID -19 patients who required long hospitalization (P < 0.01), and the blood group A was more frequent in patients with severe COVID-19 infections compared to the normal population (0.017) [10]. Two different studies have also demonstrated the possible protective effect of the blood group O [7,9,].

The relationship between ABO blood groups and cardiovascular diseases was well established previously [11]. It is known that thrombotic risks decrease significantly in blood group O compared to the non-O [11, 12]. Studies have shown that the micro thrombosis developing in the COVID-19 infection in pulmonary vascular bed lead to a serious contribution in the acute respiratory syndrome; therefore, the use of prophylactic anticoagulants was included in the guidelines, as well [13,14]. There are opinions arguing that the protective effect demonstrated in blood group O is based on this phenomenon [15]. The present study, there was no significant relationship between the clinical outcomes and the blood groups. However, this is an important study amongst Turkish COVID-19 patients that demonstrate the blood group A might be susceptible to COVID-19 infection and the blood group O might be somewhat protective which should be better elucidated in prospective much larger studies, as well.

The limitation of this study is the somewhat small number of patients with the clinical outcomes may have caused the failure in demonstrating the effect of blood groups on clinical outcomes in statistical terms. 

In conclusion, the present study demonstrate that the blood group O might be protective while the blood group A might have increased susceptibility to the disease, and guidelines set forth by the Centers for Disease Control and Prevention (CDC), WHO and Healthy authorities, such as social distancing, hand hygiene, mask use strictly should be followed. One might conclude that stronger, stringent measures should be taken when taking care of individuals with the blood group A for possible COVID-19, i.e. SARS-CoV-2 infection susceptibility. There is a need for further molecular studies to elucidate the relationship between the blood groups and the disease. Prospective larger multicentre studies may be needed in order to further elucidate the role of possible somewhat protective role of the blood group O.
